# A Context-Sensitive Active Contour for 2D Corpus Callosum Segmentation

**DOI:** 10.1155/2007/24826

**Published:** 2007-12-31

**Authors:** Qing He, Ye Duan, Judith Miles, Nicole Takahashi

**Affiliations:** ^1^Department of Computer Science, College of Engineering, University of Missouri-Columbia, Columbia, MO 65211, USA; ^2^Thompson Center for Autism, University of Missouri-Columbia, Columbia, MO 65211, USA

## Abstract

We propose a new context-sensitive active contour for 2D corpus callosum segmentation. After a seed contour consisting of interconnected parts is being initialized by the user, each part will start to deform according to its own motion law derived from high-level prior knowledge, and is constantly aware of its own orientation and destination during the deformation process. Experimental results demonstrate the accuracy and robustness of our algorithm.

## 1. INTRODUCTION

Since the seminal work of [1], deformable models have achieved great success in various areas of visual computing such as computer vision and image processing. They have become one of the dominant techniques in medical image segmentation [2]. Researchers however have not yet succeeded in developing completely automatic segmentation techniques that can incorporate high-level prior information of shape, position, orientation, symmetry, landmarks, as well as image intensity and texture characteristics to achieve segmentation accuracy and repeatability [3]. One of the challenges is that current deformable models have little-to-no explicit “awareness” of where they are in the image, how their parts are arranged, or to what structures they or any neighboring deformable models are converging during the deformation process.

Recently, McInerney et al. introduced a novel approach for automatic medical image segmentation that combines deformable model methodologies with concepts from the field of artificial life [3]. They proposed deformable organisms that possess deformable bodies with distributed sensors, as well as brains with motor, perception, behavior, and cognition centers. Deformable organisms are perceptually aware of the image analysis process. Their behaviors, which manifest themselves in voluntary movement and alteration of body shape, are based upon sensed image features, prestored anatomical knowledge, and a deliberate cognitive plan.

Inspired by the work of [3], in this paper, we introduce a new part-based, context-sensitive active contour for 2D corpus callosum segmentation from MR images. Unlike the fully automatic approach of [3], our method is semiautomatic. It requires the user to interactively initialize a seed contour (through as little as three mouse clicks) that consists of four interconnected parts. Each part of the seed is aware of its own orientation, its target structure, and more importantly can have its own motion law tailored for its corresponding target structure. By allowing the user to interactively initialize the model, our algorithm has a much lower computational complexity compared with the full automatic approach of [3].

The rest of this paper is organized as follows. In Section 2, we briefly introduce the background knowledge of our work. In Section 3, we present our method in detail, including initialization, contour evolution, and fornix elimination. Experimental results and validation are shown in Section 4 with some discussions. Section 5 concludes the paper.

## 2. BACKGROUND

### 2.1. Corpus callosum segmentation

Corpus callosum (CC) is the major communication pathway between the two cerebral hemispheres and mainly consists of axons. It is responsible for conduction of signals between homologous and heterotopic cortical regions and is an essential component for brain lateralization and interhemispheric communication. Structural changes, such as size and shape changes in the corpus callosum occur in a variety of neurological diseases, such as dyslexia, schizophrenia, autism, and bipolar and unipolar disorders. Therefore, neurologists are interested in looking at the corpus callosum and analyzing its shape. Magnetic resonance imaging (MRI) is regarded as the best method to obtain cross-sectional area and shape information from corpus callosum.

Although segmenting the CC seems simple, it turns out to be nontrivial. The global shape of the CC is relatively consistent, but the local shape variation is dramatic. The intensity of the CC also varies from one image to another. There can be gaps in the boundary of the CC almost anywhere, and parts of the CC may be narrow or have bumps. The most challenging problem is the existence of the fornix, which is a thin structure that may or may not contact the CC in the midsagittal MR image. It is almost the same brightness as the CC; and the size and position of the contact region can vary considerably. Because of these challenges, traditional active contour models will not be robust enough to correctly extract the boundary in many cases. Manual tracing of CC boundary is still the most frequently used method in clinical studies, such as in [4–6], which is time-consuming, error-prone, and operator-dependent.

### 2.2. Deformable models

Active contour models (snakes) have been widely used in image segmentation since their introduction [1]. These methods are iteratively updated according to various forces designed to seek out object/region boundaries while maintaining smoothness of the fitted contour [7]. Interactively controlled forces may also be introduced to allow the user to guide the segmentation, which made active contours particularly popular for medical imaging applications. A survey of early work of deformable models in medical image segmentation can be found in [2]. There is more recent work on snakes and their variants, such as [7–9].

The problem of most of the current active contour models is that they have little “awareness” of where they are in the image, how their parts are arranged, or to what structures they are converging [3]. Thus, there is a need to combine the low-level feature detection ability of active contour models with flexible high-level knowledge, which triggers the top-down and bottom-up combination scheme. The work in [10] proposed a method called united snakes which combined snakes and live wire [11] together. Live wire, or intelligent scissors, is an effective interactive boundary tracing tool. United snakes use live wire seed points as the hard constraints of snakes. This method can overcome a lot of disadvantages of a single live wire or snakes method. However, we have to carefully do a boundary tracing in order to get a sufficient number of seed points. The work in [12] proposed a sketch-initialized subdivision-curve snake and applied it to 2D segmentation of the CC as well as other structures. This method is rapid, accurate, and repeatable, but it requires a special pressure-sensitive device for user initialization. Deformable organism for medical image analysis was introduced in [3], where artificial life was incorporated in snakes in order to solve the “unawareness” problem. A series of routines were designed to find different parts of the object. The CC was used as an example to test their algorithm. This method was further extended by [13] which applied a physical-based implementation in order to provide an opportunity for the expert to intervene the segmentation intuitively. The results on CC segmentation in [13] showed that minor user interactions could further improve the segmentation accuracy.

Our method is mostly inspired by [3]. However, instead of using comprehensive artificial intelligence, we divide the active contour into several parts according to our prior knowledge of the segmented object. Each part of the contour is assigned to a certain part of the object, and a set of deformation rules are designed for each part, respectively. In this way, each part of the contour is aware of its destination. Furthermore, to ensure global awareness, these parts of the contour are connected by several sensor points, which are the end points of each part.

## 3. CONTEXT-SENSITIVE ACTIVE CONTOUR SEGMENTATION OF THE CORPUS CALLOSUM

**Figure 1:**
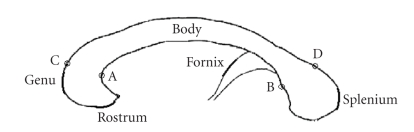
CC structure and partition of the CC boundary (image modified based on original illustration from [3]).

The anatomical structure of the CC is shown in Figure 1. Accordingly, we can roughly divide the boundary curve of the CC into 4 parts (see Figure 1), which are anterior (CA), posterior (BD), upper boundary (DC), and lower boundary (AB). Instead of using a closed-curve representation, we treat the four parts as four separate curves, which are connected by four sensor points. There are several advantages of using open-curve. First, it implicitly integrates our prior knowledge into the curve evolution process. In contrast, a closed curve cannot be aware of any high-level information after it has been initialized. Secondly, this curve partition allows us to use different parameters of active contour evolution for each part, so that each curve can better fit the curvature feature of the boundary. Thirdly, it will make fornix detection easier, since we only need to search along curve AB to find the fornix.

### 3.1. Initialization

**Figure 2:**
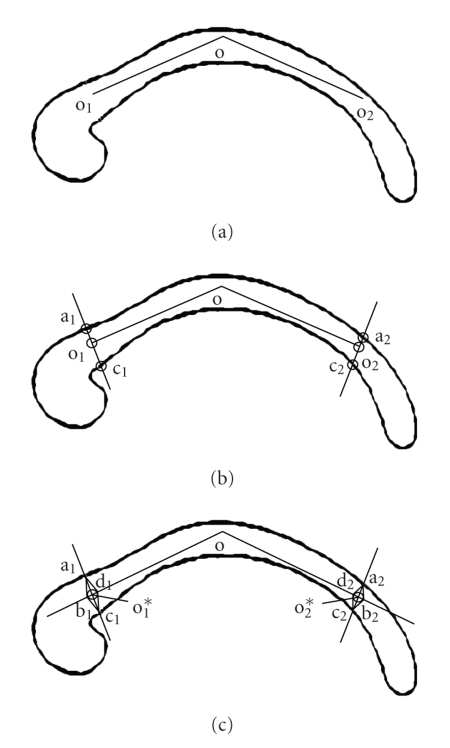
Illustration of the seed initialization process. (a) User-initialized polyline. (b) Point tracing. (c) Completed seed contour.

**Figure 3:**
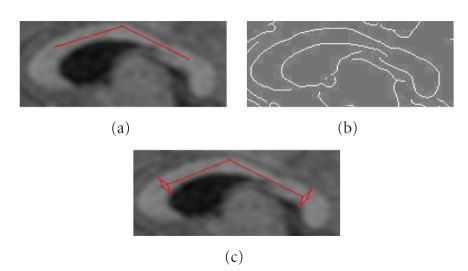
An example of the initialized seed. (a) User-initialized polyline. (b) The edge map. (c) The completed seed contour.

The initialization requires the user to click three points to form a polyline o_1_oo_2_ within the body of the CC (see Figure 2(a)). After that, a seed contour is constructed in the following fashion. First, an edge map is generated through canny edge detection. Prior to edge detection and any other operations, we perform a Gaussian smoothing to the image in order to reduce the noise. The width σ of the Gaussian function is selected so that there is no small edge in the internal area of the CC. The edge map of the smoothed image is shown in Figure 3(b).

We then do a point-tracing to find the four points on the edge (see Figure 2(b)). Starting from o_1_, we trace along a line perpendicular to line o_1_o in both directions to find edge points a_1_ and c_1_. Each one is the first point on the edge along our tracing direction. Similarly, we trace from o_2_ to find a_2_ and c_2_. In case there is a small gap, where the tracing line intersects with the edge, we may either find an edge point on the background, or never find an edge point until we reach the image boundary (the image boundary is treated as edge in our case). To avoid this problem, we trace along several lines whose angles with the original tracing line are within a small range [−θ, θ ], where θ is a positive small angle. Among the first edge point on each tracing line, the one with the smallest distance to the starting point is selected. If the gap is so large that we cannot find any edge point within [−θ, θ ], we will increase the value of θ to cover a larger search range. However, in our experiment, $\theta =20^{\circ }$ can satisfy all the images since there is usually high contrast on the upper and lower boundary of the CC.

We then show how to construct points b_1_ and d_1_ in Figure 2(c), and b_2_ and d_2_ following the same way. We assume that there is always a small neighborhood around o_1_ completely inside the CC region. This assumption is reasonable since the user can easily locate the end points in the middle with some margin to both the upper and lower boundaries. To best guarantee this assumption, we substitute o_1_ with the middle point of line segment a_1_c_1_, denoted as ${{\text{o}}_{1}}^{*}$. Thus, we can draw a circle centered at ${{\text{o}}_{1}}^{*}$ with a small enough radius (see Figure 2(c)). However, b_1_ and d_1_ are two intersection points of line o${{\text{o}}_{1}}^{*}$ and the circle. Now we have the initial seed for each part of the curve—a_1_b_1_c_1_ for anterior, c_2_b_2_a_2_ for posterior, a_2_d_2_od_1_a_1_ for upper boundary, and c_1_d_1_od_2_c_2_ for lower boundary. The four seeds are connected by four sensor points—a_1_, c_1_, a_2_, and c_2_. Figures 3a, and 3(c) show the initial polyline and the completed seed on the real image. Conceptually, this seed is a polygon with some edges overlapping in the body of the CC, while the four sensor points serve as both the separation of the entire curve and the connection of each curve segment. During the curve evolution process, these four points will not move since they are already on the boundary.

The performance of the curve evolution can be guaranteed only if the initial seed is completely inside the CC region. The seeds of left and right parts can be inside based on the above assumption. Normally, three points are sufficient to make an inside polyline. However, for some abnormal CC shapes, more points may be needed; so in our algorithm we allow the user to click as many points as needed to make the initial polyline.

### 3.2. Contour evolution

Consider a family of smooth curves *C* (*p, t* ) connecting two given end points, where *p* parameterizes the curve and *t* parameterizes the family. This family evolves according to the following partial differential equation [14]:

(1) $$\frac{\partial C(p,t)}{\partial t}=F\overset{\to }{n},\;\;C(p,0)=C^{0}(p),$$

where $\vec{n}$ is the unit normal vector of *C* (*p, t*), *F* is the speed function, and *t* can be considered as the time parameter. The speed function *F* has the form similar to that in [15]:

(2) $$F=(v+\varepsilon k)g-\gamma (\nabla g•\overset{\to }{n}),$$

where k is the curvature of the curve, v is a positive constant speed, and ε, γ are two coefficients. g is a function derived from the input image *I*; and

(3) $$\begin{array}{cc} g(x) & =\frac{1}{1+\alpha {\left(\text{NG}(x)\right)}^{2}},\\ \text{NG}(x) & =\frac{\left\|\nabla (G_{\sigma }*I)\right\|}{\max_{I}\left\|\nabla (G_{\sigma }*I)\right\|}, \end{array}$$

where NG(x) is the normalized gradient magnitude at pixel *x* of image *I*, $G_{\sigma }*I$ is the image smoothed by a Gaussian kernel, ∇ is the gradient operator, and α is a coefficient. The first term in (2) causes the curve to grow along its normal direction, and the second term acts in an opposite direction to the normal when the curve reaches the object boundary.

The orientation of the normal vector for each curve segment is specified upon initialization. We consider each curve segment as part of a closed curve drawn counterclockwise, and the normal orientation is defined as pointing outward of this closed curve. In order to get a correct normal orientation, the points on each curve should be arranged in a proper sequence.

### 3.3. Numerical integration

We employ explicit Lagrangian approach to solve the curve evolution equation. Equation (1) can be approximated numerically by

(4) $$C(p,t+\Delta t)=C(p,t)+(\Delta t)F\overset{\to }{n},$$

where Δt is a small time step. Smooth curves are represented by polygonal lines composed of vertices ${\left\{P_{i}\right\}}_{i=1}^{M}$. In order to achieve extra stability and accelerate the convergence, a tangent speed component is added in the discrete curve evolution [14]:

(5) $$P_{i}^{k+1}=P_{i}^{k}+\Delta t^{k}(F_{i}^{k}{\overset{\to }{n}}_{i}^{k}+G_{i}^{k}{\overset{\to }{t}}_{i}^{k}),$$

where $P_{i}^{k}$ indicates the location of vertex *i* in the *k*th iteration, ${\vec{n}}_{i}^{k}$ and ${\vec{t}}_{i}^{k}$ are the unit normal vector and tangent vector at $P_{i}^{k},$ respectively, and $F_{i}^{k}$ is a discrete approximation of *F*. The tangent speed component *G* is defined as

(6) $$G_{i}^{k}=\frac{d_{i}^{k}-d_{i-1}^{k}}{d_{i}^{k}+d_{i-1}^{k}},$$

where $d_{i}^{k}$ is the distance between two neighboring vertices $P_{i}^{k}$ and $P_{i+1}^{k}$.

We use the method in [16] to estimate the time step $\Delta t^{k}$:

(7) $$\Delta t^{k}=\frac{m_{e}}{M_{F}^{k}},$$

where $m_{e}$ is the unit grid cell length of the image data and $M_{F}^{k}$ is the maximum magnitude of the speed F in the Kth iteration.

There are several ways to numerically calculate curvature and normal direction of each curve point. The normal direction of a vertex is approximated by the average of the normal vectors of two incident line segments. We use circular approximation in [14] to calculate curvature. Suppose * A, O, B* are successive points on a curve, and we want to calculate the curvature of *O*. The circular approximation is

(8) $$k=\frac{4S}{abc},$$

where *a = |AO|, b = |BO|, c = |AB|*, and * S* is the area of triangle *ABO*. We assign a sign to the curvature of each point. With the normal pointing outwards, the convex point has a negative curvature and the concave point has a positive curvature.

All vertices are labeled active at the beginning. Vertex *i* terminates its motion when $F_{i}^{k}$ is sufficiently small [14], and it is then labeled as inactive. When all vertices are inactive, the curve evolution stops.

### 3.4. Curve regularity

To ensure that the numerical simulation of the curve evolution proceeds smoothly, we must maintain the regularity of the curve. There are two issues to concern: point density and self collision.

To maintain proper density of the curve points, a new vertex is inserted between two adjacent vertices if the distance between them is bigger than the maximum edge length, and a vertex is deleted if its distance with one of its neighbors is smaller than the minimum edge length. The inserted vertex is initially marked active. The proper edge length should be chosen so that the curve points are dense enough to capture the details of the shape, while overhead points are avoided to reduce computational cost. In our case, the maximum edge length is 4 and the minimum edge length is 1, with the length of the CC ranges between 130–160 (all in pixel unit).

To avoid self collision within each part of the curve, we apply a collision detection technique. Our method is similar to that in [16] while more efficient in convergence. If the distance between two nonadjacent vertices is smaller than the minimum edge length, we connect the two vertices and delete all the vertices in between.

### 3.5. Fornix removal

**Figure 4:**
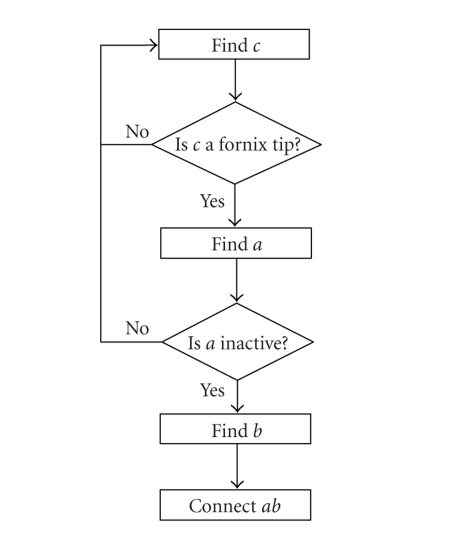
Flow chart of the fornix removal algorithm.

**Figure 5:**
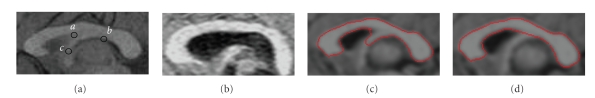
(a) Distinct points around the fornix. (b) The example of disconnected fornix. (c) The result without fornix removal. (d) The result with fornix removal.

The fornix may or may not contact the CC in midsagittal and surrounding slices. It is a thin structure with similar intensity level as the CC. For this reason, a standard active contour model cannot extract the correct CC boundary connected to fornix. The work in [3] applied a find-fornix routine based on the parallelism between the lower boundary and the medial axis. Lee et al. [17] used a heuristic method to detect the feature points of the fornix. Compared with their methods, ours is much simpler. Since we know the fornix always appears beneath the body of the CC, we only need to search along the lower boundary to detect it. As shown in Figure 5(a), there are three distint points around the fornix. If we find points *a* and *b*, we can connect them to get rid of the fornix tip. By carefully studying the curvature characteristics around the fornix, we design the following fornix removal algorithm. The flow chart of the algorithm is shown in Figure 4.

1. *Find c*: Search along the lower boundary curve segment to find the fornix tip (point * c* ), which is the point with the smallest curvature.
2. *Check c*: if *curvature ratio* is bigger than a threshold, continue the next step; otherwise, go back to *Find c*.
3. *Find a*: Search between point *c* and the left end of this curve to find the left corner of the fornix (point * a* ), which has the biggest curvature.
4. *Check a*: If point *a* is active, go back to *Find c*; otherwise, continue the following steps.
5. *Find b*: Search between point *c* and the right end of the curve, and find point *b* such that the normal direction of point *b* is perpendicular to the line from point *a* to point *b*.
6. *Connect ab*: Connect point *a* and point * b*, resample points between *a* and *b* to maintain the curve regularity, and mark the vertices between *a* and *b* inactive.

In the above algorithm, the curvature is a signed value with the curvature sign defined in 3.3. In Step 2, *curvature ratio* is the absolute value of the curvature of *c* divided by the absolute value of the average curvature along the curve. In the case of Figure 5(a), this ratio is much bigger than one because the curvature of the fornix tip is conspicuously smaller than other points on the curve. However, there may be the case that the fornix is not connected to the CC, such as in Figure 5(b). Since there is no distinct fornix tip along this curve, *Find c* routine in the initial algorithm may find an arbitrary point which happens to have the smallest curvature. In this case, the *curvature ratio* could not exceed a threshold due to the smoothness of the curve, and the algorithm will never do anything to the curve since it is always looped in *Find c*.

Since the fornix tip will not appear until after a certain time of active contour evolution, if we started removing it too early, what we found may not be the real fornix. Step 4 is to make sure it is the proper time to remove the fornix.

The resampling process in *Connect ab* introduces new vertices between * a* and * b*, which would begin their motion toward the fornix tip again. We mark them inactive so that they will not form a new fornix after the end of the algorithm.

The results with and without the fornix are shown in Figures 5(c), and 5(d).

## 4. EXPERIMENTAL RESULTS AND DISCUSSION

### 4.1. Parameter setting

There are several parameters in (2) and (3). One advantage of our method is that we can use different parameters for different parts of the CC boundary. We find the four parts have different image features as well as curvature features. For example, the anterior and posterior curves have more high-curvature regions than the other two curves, and the upper boundary has more reliable edge than the lower boundary. Accordingly, ε should be larger for the middle parts and smaller for the left and right parts, and γ should be larger for the lower boundary and smaller for the upper one. In our experiment, the parameters in (2) and (3) fall into the following ranges: v=2∼5, ε=0.5∼2, γ=7∼15, α=100∼500, σ=2∼3 for Gaussian smoothing of the image. The threshold of the curvature ratio in 3.6 is set to 10 in our experiment.

### 4.2. Segmentation results

We perform our algorithm on 2D brain MR images from different subjects. The experiment is running on an Intel(R) Pentium(R) D (2.8 GHz) PC with Windows Vista. The total time for one MR image is 2-3 seconds.

**Figure 6:**
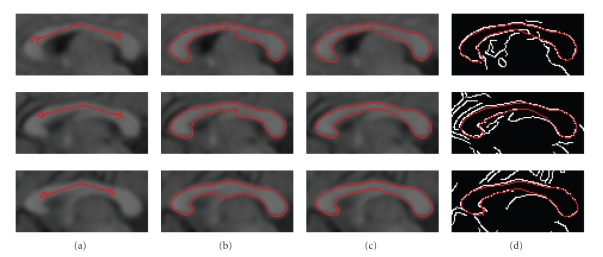
(a) The seed contour initialized by the user. (b) The fornix begins to appear without fornix removal. (c) The results with fornix removal. (d) The results overlaid on the edge map.

Figure 6 shows the result on different MR slices of the same subject. The first column shows the user-initialized seed, the second column shows the fornix tip beginning to appear without fornix removal mechanism, the third column shows the results with the fornix removed, and the last column shows our result overlaid on the edge map. As we can see in Figure 6, the shapes of the CC look similar, but the image features at the boundaries as well as the fornix differ from one slice to another. Even if there is no explicit fornix on the image, there still might be a gap where the fornix is located, thus causing a dip of the lower boundary curve. As shown in Figure 6(d), our results can match the edge map accurately and bridge the gaps.

**Figure 7:**
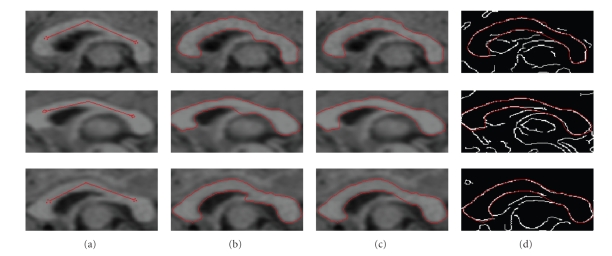
(a) The seed contour initialized by the user. (b) The fornix begins to appear without fornix removal. (c) The results with fornix removal. (d) The results overlaid on the edge map.

Figure 7 shows the results on different subjects. The four columns are the same as in Figure 6, and each row represents the midsagittal slice of a different subject.

### 4.3. Validation

We use the measurements in [15, 18, 19] to quantitatively evaluate our segmentation results. We denote the correct segmentation result as $C_{\text{true}}$, our segmentation result as $C_{\text{seg}}$, and |•| as the area enclosed within the result. The following measurements are calculated.

1. False negative fraction (FNF), which indicates the fraction of structure included in the true segmentation but missed by our method:
$\text{FNF}=|C_{\text{true}}-C_{\text{seg}}|/|C_{\text{true}}|.$
2. False positive fraction (FPF), which indicates the amount of structure falsely identified by our method as a fraction of true segmentation:
$\text{FPF}=|C_{\text{seg}}-C_{\text{true}}|/|C_{\text{true}}|.$
3. True positive fraction (TPF), which indicates the fraction of the total amount of structure in the true segmentation that is overlapped with our method:
$\text{TPF}=|C_{\text{seg}}\cap C_{\text{true}}|/|C_{\text{true}}|.$
4. Dice similarity:
$\text{Dice}=2\times |C_{\text{seg}}\cap C_{\text{true}}|/(|C_{\text{true}}|+|C_{\text{seg}}|).$
5. Overlap coefficient:
$\text{overlap}=|C_{\text{seg}}\cap C_{\text{true}}|/|C_{\text{true}}\cup C_{\text{seg}}|.$

The last two measurements range between 0-1 with one indicating a perfect agreement between $C_{\text{true}}$ and $C_{\text{seg}}$. The overlap measurement is a stronger test than Dice similarity for segmentation accuracy [18].

We use the results of manual segmentation by a trained expert as the ground truth ($C_{\text{true}}$), and compare our results ($C_{\text{seg}}$) with the ground truth. The time for an experienced expert to segment the CC on one slice is about 20 minutes. The experiment is performed on MR images of 16 subjects, and the mean and standard deviation of each measurement across subjects are listed in Table 1. The table shows low FNF and FPF, and high values in the other three measurements. This result indicates our method has high accuracy.

### 4.4. Initialization issue

**Figure 8:**
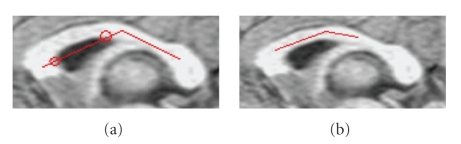
(a) The portion between two circles is outside. (b) The seed not covering the fornix.

We have designed some generic principles for the seed initialization. First, as stated in 3.1, the seed should be roughly within the body of the CC and not extend to the anterior and posterior parts. If the seed extends deep into the anterior or posterior part, the final curve will not be able to cover the whole anterior or posterior part. Second, the initial seed cannot have any portion outside the CC (see Figure 8(a)). This is because the normal orientation of each curve is predefined given that they are all inside the CC. To ensure that the seed is totally inside the CC, we allow the user to click more than three points if needed. Third, the length of the seed should cover the fornix gap, which means the right endpoint should be on right of the fornix (see Figure 8(b)). This is to make sure that the fornix tip only appears on the lower boundary curve.

These principles are generally quite easy to follow. Since users can recognize the body of the CC as well as the fornix, they are able to locate the seed within the body and cover the fornix span without difficulty.

## 5. CONCLUSION

In this paper, we have proposed a context-sensitive active contour method for 2D corpus callosum segmentation. This method takes advantage of prior knowledge by dividing the active contour into several parts and connecting them by sensor points. After a simple user initialization, a set of rules derived from prior knowledge will complete the initialization and guide the model deformation subsequently. The challenging problem caused by the fornix has been successfully solved. Experimental results demonstrate our method is fast, accurate, and easy to operate.

For future work, we plan to incorporate recognition into our segmentation framework to further reduce the user interaction by automatically locating the initial points in each part of the object, as well as recognize the connectivity of the fornix to the CC. Furthermore, although this algorithm is designed specifically for the corpus callosum, we will investigate the possibility of applying the general principle of context-sensitive active contour for segmenting other brain structures as well.

## Figures and Tables

**Table 1:** Quantitative validation results.

	FNF	FPF	TPF	Dice	overlap
Mean	0.0689	0.0613	0.9525	0.9364	0.8803
Std	0.0613	0.0401	0.0438	0.0178	0.0304
